# Prefrontal GABA and glutamate–glutamine levels affect sustained attention

**DOI:** 10.1093/cercor/bhad294

**Published:** 2023-08-10

**Authors:** Hirohito M Kondo, Hiroki Terashima, Ken Kihara, Takanori Kochiyama, Yasuhiro Shimada, Jun I Kawahara

**Affiliations:** Department of Psychology, School of Psychology, Chukyo University, Nagoya, Aichi 466-8666, Japan; Human Information Science Laboratory, NTT Communication Science Laboratories, NTT Corporation, Atsugi, Kanagawa 243-0198, Japan; Department of Information Technology and Human Factors, National Institute of Advanced Industrial Science and Technology (AIST), Tsukuba, Ibaraki 305-8566, Japan; Brain Activity Imaging Center, ATR-Promotions, Seika-cho, Kyoto 619-0288, Japan; Brain Activity Imaging Center, ATR-Promotions, Seika-cho, Kyoto 619-0288, Japan; Department of Psychology, Hokkaido University, Sapporo, Hokkaido 060-0810, Japan

**Keywords:** sustained attention, continuous performance task, prefrontal cortex, GABA, Glx

## Abstract

Attention levels fluctuate during the course of daily activities. However, factors underlying sustained attention are still unknown. We investigated mechanisms of sustained attention using psychological, neuroimaging, and neurochemical approaches. Participants were scanned with functional magnetic resonance imaging (fMRI) while performing gradual-onset, continuous performance tasks (gradCPTs). In gradCPTs, narrations or visual scenes gradually changed from one to the next. Participants pressed a button for frequent Go trials as quickly as possible and withheld responses to infrequent No-go trials. Performance was better for the visual gradCPT than for the auditory gradCPT, but the 2 were correlated. The dorsal attention network was activated during intermittent responses, regardless of sensory modality. Reaction-time variability of gradCPTs was correlated with signal changes (SCs) in the left fronto-parietal regions. We also used magnetic resonance spectroscopy (MRS) to measure levels of glutamate–glutamine (Glx) and γ-aminobutyric acid (GABA) in the left prefrontal cortex (PFC). Glx levels were associated with performance under undemanding situations, whereas GABA levels were related to performance under demanding situations. Combined fMRI–MRS results demonstrated that SCs of the left PFC were positively correlated with neurometabolite levels. These findings suggest that a neural balance between excitation and inhibition is involved in attentional fluctuations and brain dynamics.

## Introduction

Sustained attention is critical for adaptive behaviors, such as monitoring a screen or listening to a lecture. In contrast to the importance of sustained attention in daily life, researchers have devoted their efforts to investigating spatial and temporal aspects of transient attention, such as visual search (Wolfe 2020) and attentional blink (Dux and Marois 2009). Indeed, many studies have been conducted on vigilance decrement in terms of human factors (Warm et al. 2008), but it has been difficult to quantitatively assess errors that occur rarely over long periods. A gradual-onset continuous performance task (gradCPT) was developed to overcome this difficulty (Esterman et al. 2013). The gradCPT is more sensitive to changes in attentional states than classical vigilance tasks.

In gradCPTs, participants quickly respond to frequent Go trials and withhold responses to infrequent No-go trials. The gradCPT is similar to Go/No-go tasks that require the inhibition of predominant responses, which is regarded as an important component of central executive functions (Miyake et al. 2000; Nigg 2000; Aron et al. 2004). Go/No-go tasks are accompanied by sudden onsets of stimuli, which may capture exogenous attention (Luck et al. 2021). In contrast, gradCPTs avoid attentional capture of stimulus onsets by using overlapping stimuli. Stimuli are presented quickly and repeatedly in gradCPTs, increasing the temporal pressure. Thus, participants must continuously monitor changing stimuli to maintain gradCPT performance appropriately.

GradCPT performance is affected by arousal level and attentional allocation (Esterman and Rothlein 2019). The attentional allocation model postulates that an intermediate arousal level is optimal for task performance. Attention resources are less available at a low arousal level. At a high arousal level, attention resources are sufficient, but may be allocated to task-unrelated processing, as well as task-related processing. On the basis of the Yerkes–Dodson law, these situations result in an inverted U-shaped function of task performance. For successful performance, task-related processing should be facilitated under relatively undemanding situations, whereas task-unrelated processing should be suppressed under highly demanding situations.

Arousal levels are modulated by the locus coeruleus–noradrenergic (LC–NA) system that is connected to the central executive network (Lenartowicz et al. 2013; Unsworth and Robison 2017). The LC–NA system enhances phasic neural responses to salient stimuli and suppresses responses to background noise (Kihara et al. 2015; Unsworth et al. 2018). Functional magnetic resonance imaging (fMRI) studies have demonstrated that brain networks related to vigilant attention include the frontal, parietal, and subcortical areas (Langner and Eickhoff 2013). However, findings of attentional allocation have been mixed. For gradCPTs, the dorsal attention network, including dorsal parts of the frontal and parietal cortices (Corbetta and Shulman 2002), was activated during erratic responses, whereas the default mode network, including medial parts of the frontal and parietal cortices (Fox et al. 2005), was activated during stable responses (Esterman et al. 2013; Esterman, Reagen, et al. 2014; Fortenbaugh et al. 2018). In contrast, another study using multi-voxel pattern analysis found that activations in the dorsal attention and default mode networks did not distinguish attentional states (Rosenberg et al. 2015). Thus, different approaches are needed to examine neural mechanisms of sustained attention.

We used magnetic resonance spectroscopy (MRS) to investigate brain–behavior relationships from an excitation–inhibition perspective. It is fundamentally difficult to determine whether changes in brain activity are derived from excitatory or inhibitory factors of recruited neural networks (Logothetis 2008; Kondo et al. 2018). MRS can assess in vivo measures of glutamate–glutamine (Glx) and γ-aminobutyric acid (GABA) levels in voxels of interest (VOIs). Roughly 80% of neurons in the cerebral cortex are excitatory glutamatergic, whereas the remaining 20% are inhibitory GABAergic (Rubenstein and Merzenich 2003). Blood-oxygen-level-dependent (BOLD) signals are associated with neuronal activity, which is regulated by glutamate and GABA synthesis (Logothetis et al. 2001; Buzsáki et al. 2007). This MRS study measured Glx and GABA levels during a resting state. We examined how an excitation–inhibition balance of neurometabolites is involved in individual differences in sustained attention.

We introduced 2 types of gradCPTs with different task demands to assess the attentional allocation model. Although performance is better for the visual gradCPT than for the auditory gradCPT, sustained attention fluctuates within a similar temporal range, regardless of sensory modality (Terashima et al. 2021). Based on this model, we hypothesized that Glx levels are important to enhance task-related processing in the less demanding visual gradCPT, whereas GABA levels are critical to inhibit task-unrelated processing in the highly demanding auditory gradCPT. An MRS study demonstrated that performance of Go/No-go tasks was influenced by the balance of Glx and GABA levels in the left prefrontal cortex (PFC) (Koizumi et al. 2018). Another study showed that participants with high GABA levels in the left PFC have small magnitudes of attentional blink, i.e. reduction of attentional deficit (Kihara et al. 2016). These findings indicate that the glutamatergic or GABAergic system of the central executive network is related to task performance in serial visual presentation. We chose the left PFC as a VOI to compare the results of sustained attention with those of selective attention. The precuneus (PCu) was also chosen as a VOI because fMRI studies have revealed that the default mode network is associated with stable responses during the visual gradCPT (Esterman et al. 2013; Esterman, Rosenberg, & Noonan, 2014; Fortenbaugh et al. 2018).

In the present study, participants were scanned with fMRI while performing auditory and visual gradCPTs. We used several fMRI analyses to examine different aspects of sustained attention. Brain activity during intermittent responses in the gradCPTs was compared with brain activity related to attentional fluctuations. Common activations of auditory and visual gradCPTs were identified to probe general principles of sustained attention beyond sensory modalities. Combined fMRI–MRS analyses were conducted to clarify whether BOLD responses and neurometabolite levels are interdependent. In summary, we investigated mechanisms underlying sustained attention at behavioral, neurochemical, and neuroimaging levels.

## Materials and methods

### Participants

Twenty-nine participants (15 males and 14 females; mean ± SD, age = 25.5 ± 4.4 years, range 20–35 years) were recruited for the experiment. They were right-handed, healthy, Japanese people with normal hearing and normal vision. According to a priori power analyses with a power of 0.8 (*ɑ*-level = 0.05), we required at least 29 participants to detect significant correlations (*r* = 0.5; bivariate normal model). The present study was approved by the Research Ethics Committee of Chukyo University (approval no. RS20-017) and the Safety Committee of ATR-Promotions (approval no. AN21-056). Experimental procedures were implemented in accordance with Ethical Guidelines for Medical and Biological Research Involving Human Subjects. All participants gave written informed consent after experimental procedures were fully explained to them. They were paid for participation.

### Behavioral tasks

Participants performed auditory and visual gradCPTs in the fMRI session. The sequence of these gradCPTs was randomized across participants. We managed stimulus presentation and response collection using Presentation software (Neurobehavioral Systems, Berkeley, CA, USA). To familiarize participants with each task, practice trials preceded test trials.

**Fig. 1:**
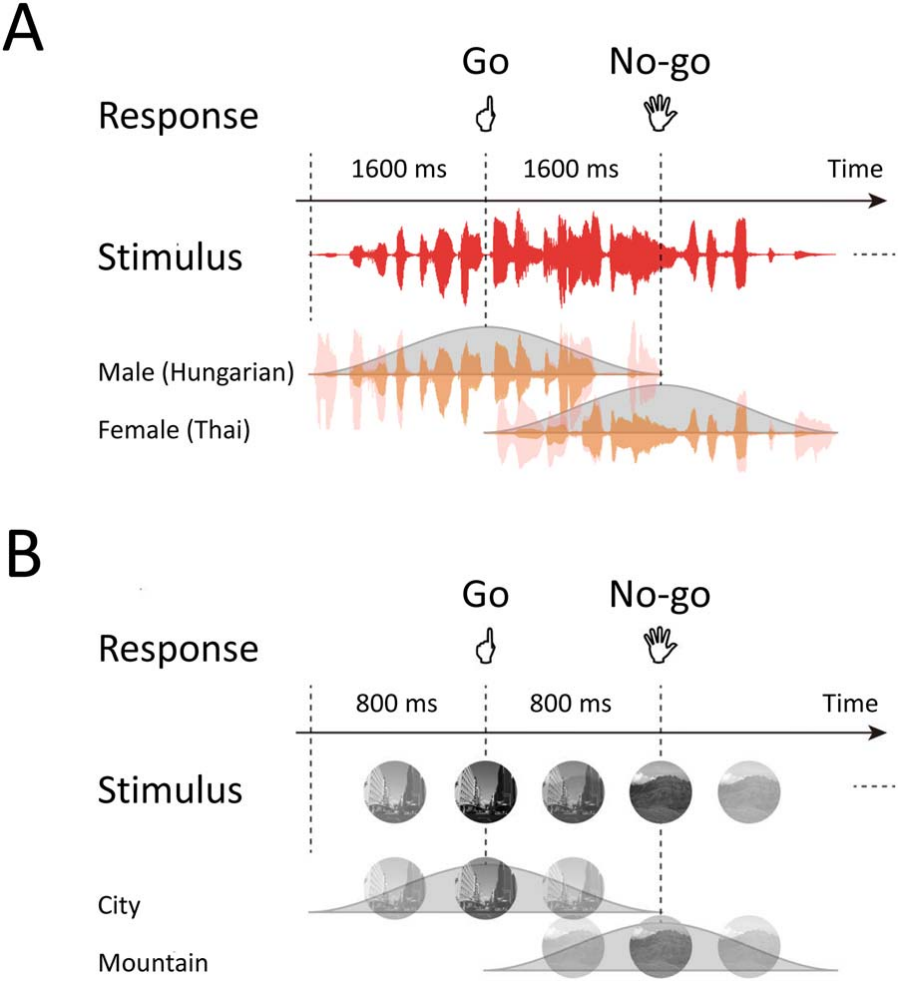
Schematic representation of gradCPTs. Stimuli temporally overlapped. (A and B) Participants judged the genders of voices in the auditory gradCPT, whereas they judged types of scenes (city or mountain) in the visual gradCPT.

For the auditory gradCPT, stimuli consisted of sequential male (90%) and female (10%) narrations that gradually changed from one to the next (Terashima et al. 2021; Kondo et al. 2022). Narrations of 10 males and 10 females were chosen from a language database, not including Japanese, and randomly presented throughout a run (Fig. 1A). Thus, using acoustic clues about stimuli, participants judged whether a voice was male or female for each trial. The sound pressure level of all narrations was adjusted to a comfortable listening level. Stimuli were delivered through plastic tubes and headphones (Hitachi Advanced Systems, Yokohama, Japan). For the visual gradCPT, stimuli were round, grayscale photographs (visual angle, 5.0°) of 10 city scenes and 10 mountain scenes (Esterman et al. 2013). Scenes were randomly presented with cities (90%) and mountains (10%). The scenes changed continuously one after another (Fig. 1B). No stimuli were repeatedly presented for either task.

Participants were instructed to press a button for Go trials (male or city) as quickly and accurately as possible and to withhold responses for No-go trials (female or mountain). They responded with their left index fingers to avoid affecting activity of language areas in the left hemisphere. A response deadline was implicit in the tasks because the current stimulus was replaced by the next stimulus with stimulus onset asynchrony (SOA). Each gradCPT comprised 4 400-s runs. SOAs were 1,600 ms for the auditory gradCPT and 800 ms for the visual gradCPT, resulting in 250 trials and 500 trials for each run.

### Imaging data acquisition

This experiment consisted of MRS and fMRI sessions. For all participants, data acquisition timing was fixed between 1:00 and 2:30 p.m. to minimize confounding effects of the circadian rhythm on MR spectra. We scanned participants on a 3-T MRI scanner (MAGNETOM Prisma, Siemens) with a body coil as a transmitter and a 20-channel head coil as a receiver. We placed small, comfortable, elastic pads on both sides of a participant’s head to minimize head motion.

**Fig. 2:**
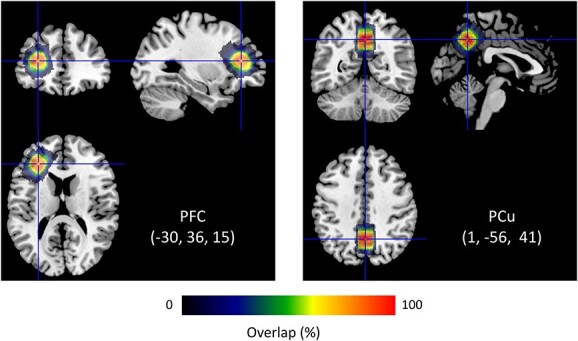
Overlapping of MRS voxels obtained from participants (*n* = 29). Centroids of voxels are positioned in MNI coordinates (−30, 36, 15) for the left prefrontal cortex (PFC) and (1, −56, 41) for the precuneus (PCu). Each voxel contains gray matter, white matter, and cerebrospinal fluid: 38.1%, 59.3%, and 2.6% for the PFC; 69.5%, 11.4%, and 19.1% for the PCu.

MRS sessions were conducted before fMRI sessions to avoid gradient-induced frequency drift (Harris et al. 2014). A session included 2 runs for the distinct 20 × 20 × 20 mm^3^ voxels that were located in the left PFC and PCu (Fig. 2). The PFC voxel was positioned at the anterior part of the middle frontal gyrus (Brodmann area: BA 46), whereas the PCu voxel was centered bilaterally on the interhemispheric fissure (BA 7). Specifically, the PFC voxel was individually tilted to maximize gray matter (GM) volume and minimize white matter (WM) and cerebrospinal fluid (CSF) volumes. Using a FASTEST map sequence (Gruetter 1993), we performed manual shimming (5–10 min) of the magnetic field in the voxel to avoid line broadening. We used the MEGA-PRESS technique (Mescher et al. 1998) to obtain GABA-edited spectra from single-voxel acquisitions: repetition time (TR) = 1,500; echo time (TE) = 68 ms; 384/64 measurements, i.e. 192/32 on–off pairs, with/without water suppression; spectral bandwidth of 2 kHz with a sampling rate of 2,048 points; editing pulses applied at 1.9 ppm (edit-on) and 7.5 ppm (edit-off). Due to co-edited macromolecule contamination, we assessed GABA+. An MRS session lasted 40 min.

fMRI sessions consisted of 4 410-s runs for auditory and visual gradCPTs. For each run, we acquired 205 volumes using the multi-band, gradient-echo echo-planar imaging (EPI) sequence. Functional images sensitive to the BOLD response covered the whole brain: 72 consecutive slices parallel to the plane of the anterior–posterior commissure. A T2*-weighted EPI sequence was used with the following parameters: TR/TE = 2,000/30 ms; flip angle = 80°; multiband acceleration factor = 3; partial Fourier = 6/8; matrix size = 100 × 100; number of slices = 72; slice thickness = 2 mm, no gap, interleaved acquisition; voxel size = 2 × 2 × 2 mm^3^. At the beginning of an fMRI session, we acquired a B0 field map to correct for geometric distortions: TR/TE1/TE2 = 750/5.17/7.63 ms; flip angle = 50°; matrix size = 100 × 100; 72 slices in the same orientation and geometry as the EPI sequence. An fMRI session took 40 min.

For structural MRI, we acquired 3D anatomical images of the whole brain with a T1-weighted magnetization-prepared rapid gradient echo (MPRAGE) sequence: TR/TE = 2,250/3.06 ms; inversion time = 900 ms; flip angle = 9°; 208 sagittal slices; matrix size = 256 × 256 mm; isotropic voxel size = 1 × 1 × 1 mm^3^.

### Behavioral data analysis

For gradCPTs, we defined an RT as the relative time from stimulus onset to a key press (Esterman et al. 2013; Terashima et al. 2021). The time window for the stimulus was set from 70% of the appearance phase to 40% of the disappearing phase. We assigned all key presses in the time window to the current trial. We used the shortest RT for a subsequent analysis when finding multiple key presses in a single trial. Ambiguous key presses were considered as responses to an adjacent trial. On the basis of the response assignment, we classified all trials as hit, miss, false alarm (FA), and correct rejection (CR) trials. In this case, FA trials refer to incorrect responses to No-go stimuli, whereas CR trials refer to successful inhibition to No-go stimuli. RTs for miss and CR trials without responses were linearly interpolated by estimating RTs of 2 adjacent trials. For each participant, we calculated the sensitivity (*d’*) and median RTs. We computed time-series absolute values of *z*-scored RTs in each run and termed them variance time courses (VTCs), i.e. fluctuation of sustained attention. Too quick or too slow RTs could be considered signatures of attentional fluctuation. We smoothed the VTCs with a Gaussian kernel at full width at a half maximum of 7 s.

We performed Bayes Factor (BF) hypothesis testing, in addition to classical hypothesis testing, to assess the strength of evidence for the null hypothesis (H_0_) or alternative hypothesis (H_1_) (Keysers et al. 2020). In general, BF ≥ 10 indicates strong evidence for H_1_, whereas BF ≤ 1/10 indicates strong evidence for H_0_. 10 > BF ≥ 3 indicates moderate evidence for H_1_, whereas 1/3 ≥ BF > 1/10 indicates moderate evidence for H_0_. 3 > BF > 1/3 indicates absence of evidence to support H_0_ or H_1_. We also calculated 95% credible intervals (CIs) for correlation analyses. Statistical analyses were carried out with IBM SPSS Statistics (ver. 25) and JASP (ver. 0.17.2.1) (https://jasp-stats.org/).

### MRS data analysis

Using Gannet 3.0 software (Edden et al. 2014), we analyzed MRS data as follows: zero-filling, 3-Hz exponential line broadening, and frequency and phase correction using the spectral registration. We subtracted edit-off spectra from edit-on spectra and used a Gaussian model to compute neurometabolite measures of a double Glx peak around 3.75 ppm and a single GABA+ peak at 3.00 ppm. We segmented MRS voxels into GM, WM, and CSF fractions. To correct for voxel tissue fractions, we calculated the relaxation of water signals in GM, WM, and CSF and the difference in GABA+ levels between GM and WM (Harris et al. 2015). There is still debate as to whether creatine (Cr) or water should be used as a reference (see also Supplementary Data). However, it has been suggested that water-referenced GABA+ measures are useful even in multi-site studies, with appropriate corrections (Mikkelsen et al. 2019). Finally, water-referenced Glx and GABA+ levels were estimated as neurometabolite measures with institutional units (i.u.). After spatial normalization to Montreal Neurological Institute (MNI) standard space, we computed the overlap of MRS voxels across participants using SPM12 (http://www.fil.ion.ucl.ac.uk/spm) and in-house codes, implemented in MATLAB R2020b (MathWorks, Natick, MA, USA).

### fMRI data analysis

We first preprocessed fMRI data. For each run, we discarded the 5 initial images to achieve steady-state equilibrium between radio-frequency pulsing and relaxation. After slice-timing correction, we realigned and unwarped all functional images to correct for head motion and image distortions, respectively. For distortion correction, we used a B0 field map processed using the FieldMap toolbox of SPM12 (Andersson et al. 2001; Hutton et al. 2002). The anatomical image was co-registered to the mean functional image. All functional images were normalized to MNI space, resampled to a voxel size of 2 × 2 × 2 mm, and smoothed with an isotopic Gaussian kernel of 6 mm full width at half maximum.

We entered the 4 types of trials into a design matrix and used a general linear model to perform first-level analysis (Worsley and Friston 1995). We embedded each trial type as a stick function and modeled trial-related regressors that were convolved with a canonical hemodynamic response function (HRF). We used a high-pass filter of a 128-s cut-off period to remove the artifactual low-frequency trend. Six realignment parameters were treated as nuisance covariates to remove motion effects. We calculated serial autocorrelation from pooled active voxels with a maximum likelihood procedure. The autocorrelation was used to whiten the data and the design matrix (Friston et al. 2002). Finally, we obtained FA- and CR-related contrast images for each gradCPT. We performed random-effects subtraction and conjunction analyses to identify brain activations at the population level. The statistical threshold was set at *P* < 0.05 corrected for multiple comparisons with a cluster-level family wise error.

We constructed another design matrix to examine changes in BOLD signals corresponding to the VTC, i.e. trial-by-trial RT variability. We estimated the amplitude-modulated, non-smoothed VTC that was convolved with a canonical HRF. The time-delayed VTC was downsampled to 0.5 Hz, i.e. TR = 2 s, and was used as the regressor of the design matrix. We conducted random-effects conjunction analyses using VTC-related statistical maps of the auditory and visual gradCPTs. The threshold was same as that used in the trial-based analyses.

## Results

### Behavioral results

Behavioral measures of auditory and visual gradCPTs are shown in Table 1. As we expected, paired *t*-tests revealed that hit rate, FA rate, and *d’* were better for the visual gradCPT than for the auditory gradCPT. RTs were faster for the visual gradCPT than for the auditory gradCPT. These results indicate that the auditory gradCPT, relative to the visual gradCPT, includes heavy attentional demands. However, although SOAs differed between the auditory and visual gradCPTs, the fluctuation frequency of auditory attention was similar to that of visual attention (~0.03 Hz). We found a positive correlation of fluctuation frequency, as well as of FA rate and *d’*, between the two gradCPTs. Thus, it is likely that gradCPT performance depends on intra-individual rhythms beyond sensory modalities.

**Table 1:** Auditory and visual gradCPT performance.

	Auditory gradCPT		Visual gradCPT		Difference			Correlation		
Measure	Mean	SD	Mean	SD	*t*	*P*	BF	*r*	*P*	BF
Hit (%)	74.1	14.6	96.4	0.5	7.69	<0.001	>100	0.23	0.25	0.45
False alarm (%)	23.8	11.7	18.8	10.3	2.60	0.016	4.23	0.66	<0.001	>100
Sensitivity (*d’*)	1.56	0.65	3.11	0.84	8.95	<0.001	>100	0.39	0.05	1.47
RT (ms)	1558	144	625	63	31.67	<0.001	>100	0.20	0.32	0.39
Frequency (Hz)	0.032	0.008	0.032	0.009	0.88	0.93	0.21	0.35	0.09	1.00

*Note*. gradCPTs; gradual-onset, continuous performance tasks, SD; standard deviation, BF; Bayes factor, Frequency; frequency of attentional fluctuation.

### MRS results

We obtained neurometabolite measures from MRS voxels that overlapped considerably across participants (Fig. 2). Glx and GABA+ levels (mean ± SD) were 5.54 ± 0.91 i.u. and 2.33 ± 0.90 i.u. for the left PFC; 8.17 ± 1.15 i.u. and 3.76 ± 0.84 i.u. for the PCu. Using Shapiro–Wilk tests, we determined that MRS data followed a normal distribution: *W* > 0.943, *P* > 0.15. Smirnov–Grubbs tests did not indicate any outliers of MRS data. Fitting errors (Cramér–Rao lower bounds) for Glx and GABA+ levels were 8.4 ± 2.6% and 11.2 ± 3.6% for the left PFC; 6.0 ± 1.5% and 7.9 ± 2.3% for the PCu. Thus, the quality of MRS data was satisfactory.

**Fig. 3:**
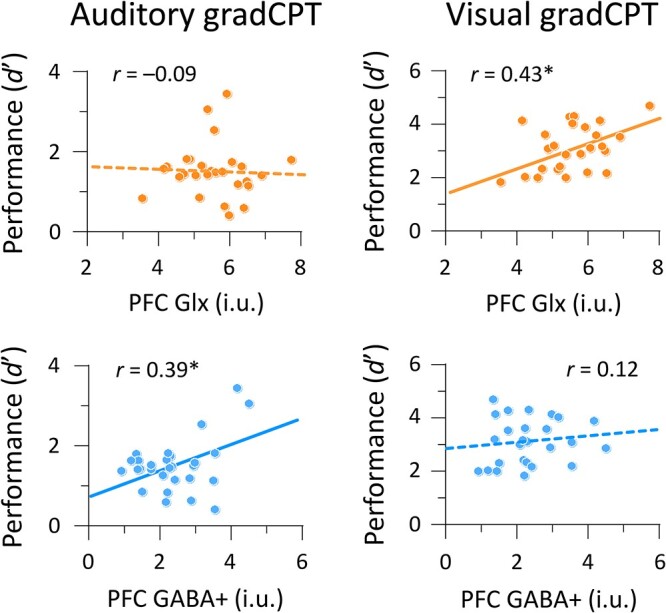
Scatterplots for the relationship between gradCPT performance and neurometabolite measures in the left PFC. Circles indicate individual data points. Solid and dashed lines represent significant and non-significant correlations, respectively. i.u.; institutional unit. ^*^P < 0.05.

**Fig. 4:**
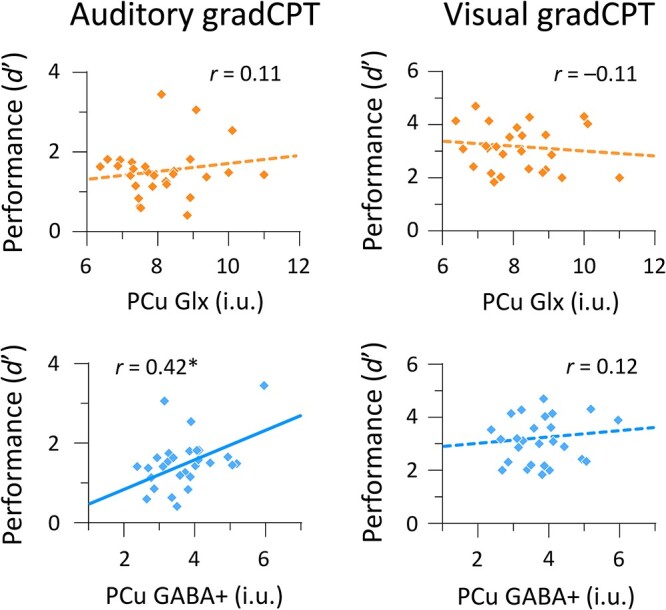
Scatterplots for the relationship between gradCPT performance and neurometabolite measures in the PCu. Diamonds indicate individual data points. Solid and dashed lines represent significant and non-significant correlations, respectively. i.u.; institutional unit. ^*^P < 0.05.

We checked brain–behavior relationships to compute correlations between neurometabolite measures and gradCPT performance (*d*’). Glx levels in the left PFC were positively correlated with visual gradCPT performance (*r* = 0.43, *P* = 0.028, BF = 2.40, 95% CI [0.04, 0.68]), but not with auditory gradCPT performance (*r* = −0.09, *P* = 0.66, BF = 0.25, 95% CI [−0.41, 0.30]) (Fig. 3, top panels). In contrast, GABA+ levels in the left PFC were positively correlated with auditory gradCPT performance (*r* = 0.39, *P* = 0.039, BF = 1.70, 95% CI [0.01, 0.64]), but not with visual gradCPT performance (*r* = 0.12, *P* = 0.55, BF = 0.29, 95% CI [−0.26, 0.46]) (Fig. 3, bottom panels). For the PCu, we did not find any significant correlation between Glx levels and task performance: *r* = 0.11, *P* = 0.57, BF = 0.28, 95% CI [−0.25, 0.45] for the auditory gradCPT; *r* = −0.11, *P* = 0.60, BF = 0.28, 95% CI [−0.45, 0.28] for the visual gradCPT (Fig. 4, top panels). GABA+ levels in the PCu were positively correlated with auditory gradCPT performance (*r* = 0.42, *P* = 0.023, BF = 2.66, 95% CI [0.05, 0.67]), but not with the visual gradCPT (*r* = 0.12, *P* = 0.56, BF = 0.29, 95% CI [−0.27, 0.46]) (Fig. 4, bottom panels). Intriguingly, GABA+ and Glx levels in the left PFC were involved in auditory and visual sustained attention, respectively. However, BF hypothesis testing did not offer strong evidence for relationships between neurometabolite measures and task performance.

### fMRI results

**Fig. 5:**
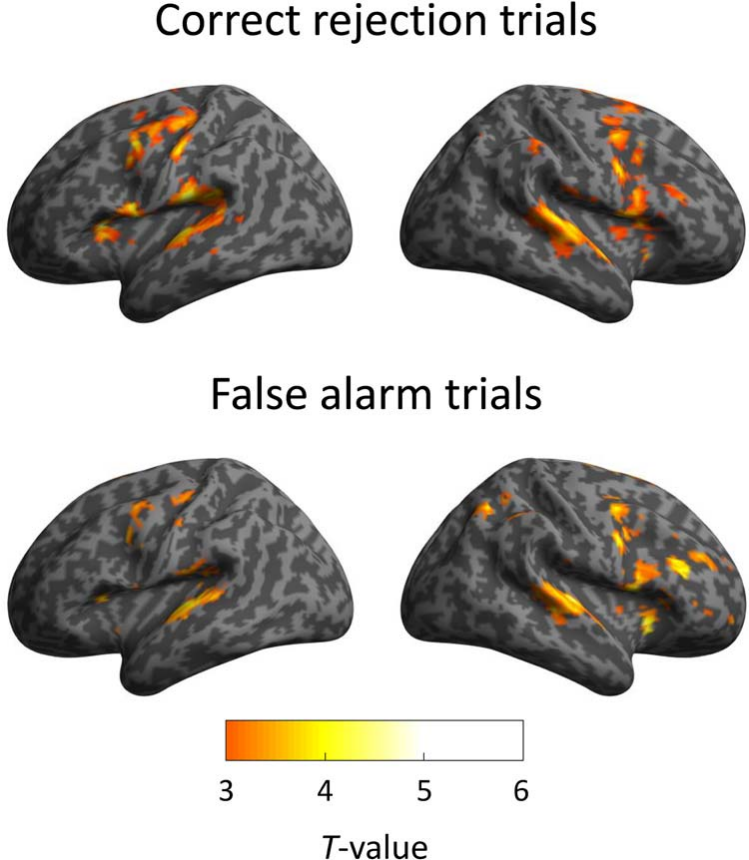
Extent and intensity of brain activity averaging auditory and visual gradCPTs (*P* < 0.05, corrected at the cluster level).

We specified brain activities in gradCPTs using an event-related analysis. Activated areas during CR trials overlapped considerably with those during FA trials (Fig. 5). We performed a cognitive subtraction analysis to compare brain activities of successful and unsuccessful inhibition. There was no significant activation in the contrast of CR-minus-FA trials. Error-specific activity was found at the anterior cingulate cortex (BA 32) in the contrast of FA-minus-CR trials. The local maximum was positioned in MNI coordinates (−20, 36, 12; *T* = 4.89). We focused on FA trials and classified these trials into auditory and visual gradCPTs. Although activations of sensory areas differed between the two gradCPTs, these tasks shared the following activated areas: the right PFC (BA 46), supplementary motor area (SMA) (BA 6), and right inferior parietal lobule (IPL) (BA 40) (Table 2). This indicates that these frontal and parietal regions, particularly the right hemisphere, are core networks of sustained attention for intermittent responses.

**Table 2:** Brain regions activated during false alarm trials in gradCPTs.

Brain region	BA	*x*	*y*	*z*	*T* value
Auditory gradCPT					
Prefrontal cortex	R46	38	50	24	4.65
Premotor area	R6	54	10	44	5.38
Supplementary motor area	R6	2	2	62	5.42
Superior temporal cortex	L22	−56	−28	10	6.63
	R22	64	−24	6	5.58
Auditory cortex	L42	−54	−26	14	5.94
	R42	56	−36	10	7.27
Inferior parietal lobule	R40	52	−36	48	4.67
Visual gradCPT					
Prefrontal cortex	R46/45	42	36	32	5.47
Anterior cingulate cortex	R32	4	36	30	4.34
Supplementary motor area	R6	6	10	60	6.25
Inferior frontal cortex	R44	52	8	36	5.72
Superior parietal lobule	L7	−28	−54	48	4.49
Inferior parietal lobule	R40	48	−54	48	4.33
Visual cortex	L19/37	−28	−82	−14	7.07
	R19/37	28	−78	−12	7.31

*Note*. Coordinates (*x*, *y*, *z*) are in Montreal Neurological Institute (MNI) stereotaxic space. BA, Brodmann area; L, left; R, right.

**Fig. 6:**
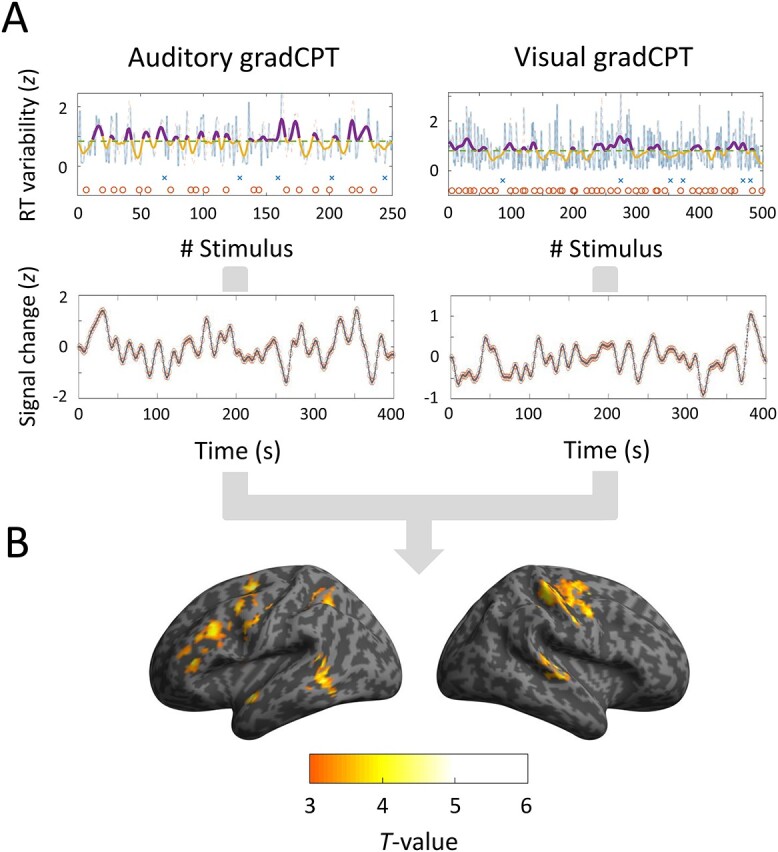
Activation areas derived from VTCs. (A) Behavioral and neuroimaging results of a representative participant. VTCs in upper panels were computed for each 400-s run in auditory and visual gradCPTs. dashed lines show median VTCs. Crosses and circles indicate FA and CR trials, respectively. VTC regressors in lower panels were convolved with a canonical hemodynamic response function and downsampled to 0.5 Hz. (B) Averaged activation areas related to the VTCs (*P* < 0.05, corrected at cluster level).

In order to examine attentional fluctuations, we computed the VTC for each run (Fig. 6A, top panels) and then produced VTC regressors convolved with an HRF (Fig. 6A, bottom panels). The pattern of VTC-related activations differed from that of event-related activations: the left IFC (BAs 45 and 44), premotor area (PMA) (BA 6), auditory-related areas (BAs 41/42 and 22), and left IPS (BA 7) (Fig. 6B and Table 3). However, there was no activation negatively correlated with VTCs. Thus, these left-lateralized, fronto-parietal areas were activated during erratic response periods, rather than during stable response periods. We used a conjunction analysis to probe a common brain activity of auditory and visual gradCPTs. These results showed activations of the left IFC (BA 45) and left PMA (BA 6) where local maxima were positioned in the coordinates (−50, 28, 22; *T* = 3.25) and (−24, −4, 56; *T* = 3.51). Thus, it is likely that left-lateralized frontal activations are closely associated with temporal changes in attentional levels, regardless of sensory modality.

**Table 3:** Brain regions activated in relation to changes in sustained attention.

Brain region	BA	*x*	*y*	*z*	*T* value
Inferior frontal cortex	L45	−42	32	12	3.94
	L44	−54	18	34	4.55
Premotor area	L6	−20	−6	50	4.62
	R6	40	−8	60	4.48
Middle temporal cortex	L21	−54	−48	8	4.39
Auditory cortex	R41/42	58	−32	10	4.49
Intraparietal sulcus	L7	−32	−52	62	3.83

### Combined fMRI–MRS results

**Fig. 7:**
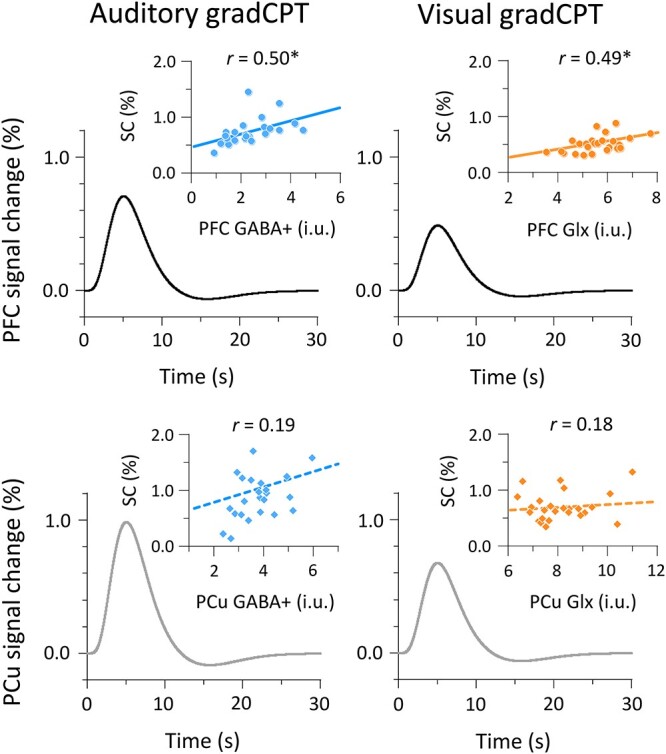
fMRI–MRS correlations in the left PFC and PCu. Curves in panels show averaged BOLD responses fitted by a hemodynamic response function. Scatter plots indicate the relationship between neurometabolite measures and SCs. Circles and diamonds in insets represent individual data points. Solid and dashed lines indicate significant and non-significant correlations, respectively. ^*^P < 0.05.

Finally, we focused on FA trials to probe the relationship between BOLD responses and neurometabolite measures. For each participant, we anatomically identified coordinates of VOIs and extracted signal changes (SCs) from the left PFC and PCu (Fig. 7). We performed a 2 (auditory vs. visual gradCPTs) × 2 (PFC vs. PCu) repeated-measure analysis of variance (ANOVA) on the SCs. The amplitude of SCs was greater for the auditory gradCPT (0.88 ± 0.40%) than for the visual gradCPT (0.61 ± 0.17%): *F*(1, 24) = 12.89, *P* = 0.001, ${\eta}_{\mathrm{p}}^2$ = 0.35. The amplitude of SCs was lower for the PFC (0.62 ± 0.14%) than for the PCu (0.86 ± 0.38%): *F*(1, 24) = 15.36, *P* < 0.001, ${\eta}_{\mathrm{p}}^2$ = 0.39. There was no interaction between task type and VOI: *F*(1, 24) = 0.67, *P* = 0.42, ${\eta}_{\mathrm{p}}^2$ = 0.03. We found positive activations in the left PFC and PCu, although event-related analyses demonstrated that these activations did not reach statistical significance. We further looked for fMRI–MRS correlations (insets in Fig. 7). For the auditory gradCPT, GABA+ levels in the left PFC were positively correlated with SCs of PFC activity: *r* = 0.50, *P* = 0.011, BF = 5.25, 95% CI [0.11, 0.73]. In the PCu, however, correlation between GABA+ levels and SCs did not reach statistical significance: *r* = 0.19, *P* = 0.36, BF = 0.37, 95% CI [−0.21, 0.52]. For the visual gradCPT, Glx levels in the left PFC were positively correlated with SCs of PFC activity: *r* = 0.49, *P* = 0.012, BF = 4.79, 95% CI [0.11, 0.72]. In the PCu, there was no significant correlation between Glx levels and SCs: *r* = 0.18, *P* = 0.39, BF = 0.35, 95% CI [−0.22, 0.51]. Consistent with brain–behavior relationships (Fig. 3), GABA+ and Glx levels in the left PFC were closely linked to BOLD responses during auditory and visual gradCPTs, respectively.

## Discussion

We illustrated fundamental principles of sustained attention using different gradCPTs. Task demands were lower for the visual gradCPT than for the auditory gradCPT, but task performance in both tasks was correlated. In addition, frequencies of attentional fluctuation were in the same range for the 2 gradCPTs. Thus, gradCPT performance is largely modulated by supra-modal attentional processes. We found positive correlations between gradCPT performance and neurometabolite measures in the left PFC. Specifically, GABA+ and Glx levels were associated with *d*’ of auditory and visual gradCPTs, respectively. The brain–behavior relationship is consistent with the attentional allocation hypothesis, in which facilitation of task-related processing is important under relatively undemanding situations, whereas suppression of task-unrelated processing is critical under highly demanding situations. We also found positive correlations between BOLD responses and neurometabolite measures in the left PFC. RT variability during gradCPTs was correlated with SCs in the left IFC and left premotor area. We will discuss behavioral, MRS, and fMRI results in turn.

Auditory and visual gradCPTs were connected by unsuccessful responses (FA rate, around 20%), rather than successful responses. From a methodological perspective, gradCPTs are similar to Go/No-go tasks that assess the ability to suppress unwanted actions or predominant responses. In the literature of sustained attention, it has been argued that the visual gradCPT includes two distinct processes: maintenance of accurate performance and strategic control for speed–accuracy tradeoff (Fortenbaugh et al. 2015). Some aspects of gradCPT performance are affected by monetary reward, but its temporal decline cannot be easily overcome by participant motivation alone (Esterman, Reagan, et al. 2014). Taken collectively, these findings suggest that a core ability for gradCPTs heavily relies on strategic control for each individual.

We found that GABA+ levels in the left PFC were positively correlated with auditory gradCPT performance. It has been proposed that the GABAergic system affects inhibition of bottom-up attention, which protects task-relevant information from task-irrelevant information (Fragopanagos et al. 2005; Olivers and Meeter 2008; Raffone et al. 2014). An MRS study demonstrated that FA rates (approximately 8%) of visual Go/No-go tasks were influenced by the Glx/GABA+ ratio in the left PFC (Koizumi et al. 2018). In addition, higher GABA+ levels in the left PFC were related to better performance during an attentional blink task (Kihara et al. 2016). This task was relatively demanding, as the accuracy of visual target detection was around 60%. Therefore, the contribution of the PFC GABAergic system to attention tasks can vary with the degree of task demand, regardless of stimulus type and sensory modality.

We also found that higher Glx levels in the left PFC were linked to fewer lapses of visual attention. The PFC glutamatergic system is probably needed to continuously maintain an alert state. However, it is not fully understood how Glx levels modulate neural energy metabolism. Activity of the right PFC during a working memory task was disrupted by ketamine, an *N*-methyl-D-aspartate glutamate receptor antagonist (Anticevic et al. 2012). In combined fMRI–MRS studies, BOLD responses to an auditory attention task were predicted by glutamate levels of the anterior cingulate cortex (Falkenberg et al. 2012) and the IPL (Falkenberg et al. 2014). This study showed that BOLD responses during the visual gradCPT were positively correlated with Glx levels in the left PFC. Thus, it is plausible that the PFC glutamatergic system can upregulate the central executive network to enhance processing efficiency of strategic control.

The right PFC, SMA, and right IPL, were activated during error-specific responses, regardless of sensory modality. Activations of the dorsal attention network observed in this study are consistent with previous findings that standard CPTs and vigilant attention tasks produce widespread frontal and parietal activations (Ogg et al. 2008; Langner and Eickhoff 2013; Rosenberg et al. 2015). A meta-analysis of fMRI studies has revealed that the SMA is closely linked to response inhibition of Go/No-go tasks (Simmonds et al. 2008). Notably, SCs in the left IFC and left PMA were correlated with moment-to-moment attentional fluctuations. A classical study postulated that the attention system has 3 major functions: (i) orienting to events, (ii) detecting signals for conscious processing, and (iii) maintaining an alert state (Posner and Petersen 1990). However, several researchers have indicated that the ability to remain alert over time, i.e. sustained attention, is not necessarily the same as the ability to quickly change to an alert state, i.e. selective attention (Petersen and Posner 2012; Tang et al. 2015; Fortenbaugh et al. 2017). Our findings support the dual-network model for top-down control (Dosenbach et al. 2007; Dosenbach et al. 2008). In this model, the fronto-parietal network initiates and adjusts adaptive control on a trial-by-trial basis, whereas the cingulo-opercular network maintains a mental set throughout an entire task epoch. Following this framework, we suggest that the dorsal and ventral attention networks are involved in analyzing error responses and maintaining attentional levels, respectively, during gradCPTs.

Intra-individual variability reflects the efficiency of attentional resources assigned to cognitive demands (Stuss et al. 2003; Klein et al. 2006). In the present study, fluctuations of attentional levels between stable- and unstable-response periods ranged from 25 to 50 s (see also Terashima et al. 2021). Temporal dynamics of attentional fluctuations differ from those of perceptual switches, which are induced by multistable stimuli, such as visual plaids (Huk and Heeger 2002) and auditory streaming (Kondo and Kashino 2009). Lapses of attention during Go/No-go tasks occurred every 15–40 s (Vaurio et al. 2009), whereas spontaneous switching between different perceptual objects ranged from several to 10 s (Pressnitzer and Hupé 2006; Kondo et al. 2012). In particular, a causal role of the PFC has long been argued in regard to perceptual switching under attentional modulation (Windmann et al. 2006; de Graaf et al. 2011). Although neural correlates of perceptual switching have been disputed, it does not seem that the PFC is implicated in initiation of perceptual organization (Paffen and Alais 2011; Kondo et al. 2018). Thus, formation and selection of perceptual objects have limited impact on gradCPT performance.

This study has some limitations. First, classical hypothesis testing showed significant correlations between gradCPT performance and neurometabolites, but BF hypothesis testing did not. Thus, future studies with larger sample sizes should test the reliability of our results. Second, VTC-related increased activations during erratic periods are consistent with previous findings on gradCPTs (Esterman et al. 2013; Rosenberg et al. 2015), but may be counterintuitive (Weissman et al. 2006). Most studies on vigilance have investigated a linear decrease in brain activity (Olsen et al. 2013). Individual-difference studies have noted that good performance in cognitive tasks induces increased brain activity (Kondo et al. 2004). Our results may be derived from over-engagement of attention to task-irrelevant information, leading to suboptimal performance (Ling and Carrasco 2006). Thus, it is possible that moderate levels of brain activity support optimal performance.

We focused on fundamental principles of sustained attention beyond sensory modality, although its importance has been overlooked in the literature. Using these gradCPTs, MRS, and fMRI, we found that the left frontal areas are responsible for maintenance of attention levels for gradCPT performance. Consistent with the attentional allocation model, our results indicate that the neural balance between excitation and inhibition is involved in modulation of sustained attention and brain activity. These findings provide new insights into an integrated understanding of sustained attention at the level of behavior, brain activity, and neurometabolites.

## Supplementary Material

Dataset_bhad294

## Data Availability

Data presented in this study are included in the supplementary material, and further inquiries may be directed to the corresponding author.
